# Investigation of Risk Factors for Pain Chronification in Patients Suffering from Infections of the Spine

**DOI:** 10.3390/jcm9124056

**Published:** 2020-12-15

**Authors:** Yina Zhao, Stefan Hemmer, Wojciech Pepke, Michael Akbar, Marcus Schiltenwolf, Ulrike Dapunt

**Affiliations:** 1 Center for Orthopedics, Trauma Surgery and Spinal Cord Injury, Heidelberg University Hospital, Schlierbacher Landstrasse 200a, 69118 Heidelberg, Germany; yinazhao_2702@hotmail.de (Y.Z.); Stefan.Hemmer@med.uni-heidelberg.de (S.H.); Wojciech.Pepke@med.uni-heidelberg.de (W.P.); Marcus.Schiltenwolf@med.uni-heidelberg.de (M.S.); 2 Clinic for Spinal Diseases, Meoclinic Berlin, Friedrichstrasse 71, 10117 Berlin, Germany; michael.akbar@meoclinic.de

**Keywords:** spinal infection, chronic pain syndrome, quantitative sensory testing (QST), multidisciplinary pain therapy, pain coping strategies

## Abstract

Background: Spinal infections represent a therapeutic challenge. The often protracted course of the disease is accompanied by pain, which can lead to a chronic pain experience even after the infectious disease has been treated successfully. The aim of this study was to investigate possible risk factors of pain chronification. Methods: In a prospective study, 14 patients with spinal infections were examined at admission (T1), at discharge from inpatient therapy (T2), and three to eight months postoperatively (T3) byquestionnaires on risk factors for pain chronification and by quantitative sensory testing (QST). Results: In-patient treatment lasted on average 45.3 days (±33.13). The patients complained of pain for 3.43 months (±2.77) prior to inpatient treatment. The visual analogue scale (VAS) for pain (0–10) and the Oswestry Disability Index detected significant improvement in the course of the study. However, patients also reported catastrophic thinking, as well as fear of movement and (re)-injury. Conclusion: In summary, our results demonstrate that patients with spinal infections did not suffer from pain chronification, but might benefit from an interdisciplinary therapeutic approach, which emphasizes promoting active pain-coping strategies, as well as addressing fear of movement and catastrophic thinking.

## 1. Introduction

Infections of the musculoskeletal system are difficult to treat and frequently require a protracted and rigorous therapy [1]. Spinal infections, or spondylodiscitis, in particular represent a therapeutic challenge [2,3] The intervertebral disc space is primarily difficult to access antibiotically, so bacteria might persist here and can be reactivated when the immune system is weakened [4]. An increase in risk factors, such as age, obesity, immunosuppression, substance abuse, chronic infections, previous spinal surgery, cardiological, metabolic or oncological pre-existing conditions, as well as a wide spectrum of pathogens, further complicate the course of the disease and call for an interdisciplinary therapeutic approach.

*Staphylococcus aureus* has been traditionally described to be the most common causative agent, however, recently also coagulase-negative *Staphylococci* and faecal bacteria have been described in association with spinal infections [2]. Furthermore, the increasing use of foreign material in the field of orthopaedics, lead to a rise in implant-associated infections [5]. Once bacteria attach to the implant surface, they embed themselves in a slimy matrix and thereby form a so-called biofilm colony. By doing so, bacteria become more resistant towards antibiotics and biocides. Therefore, biofilm formation is nowadays considered to be the most common cause for persisting infections [6,7].

Bone infections, or osteomyelitis, of the vertebral body (spondylitis) and the intervertebral disc space (spondylodiscitis) are the third most common location of osteomyelitis, after the tibia and the femoral bone. Yet, they have a rather low incidence of approximately 1:250,000 inhabitants per year [8]. Frequently, patients suffer from back and/or neck pain for a long period of time until the diagnosis of spinal infection is finally made. Ross and Fleming [9] described spinal infections as “neither common enough to be readily recognizable, nor rare enough to be a medical curiosity, [spondylodiscitis] represents a diagnostic challenge to the physician”. Therefore, spinal infections are a severely consuming disease, because patients might suffer from pain long before treatment is initiated [10]. This condition could be enhanced due to a protracted course of the disease and extensive therapy.

Furthermore, it has been demonstrated that psychological, social and also iatrogenic factors significantly contribute to chronic pain, which require an interdisciplinary therapeutic approach [11]. The impact of psychosocial factors on pain perception and the further course of a disease have been demonstrated for several chronic and oncological diseases, but the association with musculoskeletal infections has not been investigated in depth [12,13].

Therefore, we were interested in evaluating the pain sensation of patients suffering from spinal infections during the course of the disease. We hypothesized that these patients are at high risk of developing a chronic pain syndrome, which should be addressed in an interdisciplinary way early on. In order to evaluate risk factors for pain chronification, as well as coping strategies, patients who suffered from spinal infections were examined by various questionnaires. Furthermore, quantitative sensory testing (QST) was performed on all patients. This method represents a subjective psychophysical examination to quantify functions of the somatosensory system. By means of thermal and mechanical stimuli, not merely loss of function, but also gain of sensory functions can be evaluated, along with hyperalgesia, allodynia und hyperpathia [14,15,16,17].

## 2. Materials and Methods

### 2.1. Patients

A total of 14 persons were recruited for this study (prospective cohort study). The study was approved by the local ethics committee of Heidelberg University (S-466/2014). The patients were recruited using the internal patient management system of the Heidelberg University Hospital. Written and informed consent was obtained from all participants. All patients were diagnosed with spondylodiscitis (*n* = 12) or spinal implant-associated infections (*n* = 2) of the thoracic or lumbar spine by evaluating C-reactive protein (CRP) concentration, plain X-rays, magnetic resonance imaging (MRI) and computed tomography (CT) analysis. Twelve patients received a surgical intervention. Ten patients underwent debridement, dorsal spondylodesis with disc replacement due to instability; additionally one patient had an evacuation of an epidural abscess, and two patients received a debridement with implant-exchange. Two patients underwent non-surgical treatment, but received a CT-guided biopsy of the spine. All patients were examined at admission (time point 1, T1), at discharge from inpatient therapy (time point 2, T2) and three to eight months postoperatively (time point 3, T3). Exclusion criteria were chronic inflammatory disease of the musculoskeletal system, severe mental disorders, epilepsy, known dysfunction of the afferent nervous system (i.e., polyneuropathy), dementia, other factors affecting cooperation and/or consent of the test persons and lack of written informed consent to participate in the study. Patients under the age of 18 were excluded from this study. The present study refers to data collected from March 2019 until May 2020.

### 2.2. Questionnaires

The following questionnaires were used to determine risk factors for pain chronification: Patient Health Questionnaire (PHQ), Pain Catastrophising Scale (PCS), Relationship Questionnaire (RQ), Coping Strategies-Questionnaire (CSQ-D), Oswestry Disability Index (ODI), Rosenberg Self-Esteem Scale (RSES) and the Tampa Scale (TS). The PHQ explores underlying psychological tendencies, such as a somatoform component, depression, panic or anxiety. Catastrophizing level whilst in pain is measured with the PCS. The RQ determines the kind of attachment style and the general application to the patient’s personal life. Here, the secure, the dismissive-avoidant, the anxious-preoccupied and the fearful-avoidant style of attachment are evaluated. With the CSQ-D, the different kinds of coping strategies when experiencing pain are explored. There are active coping strategies like Reinterpreting pain sensations, diversion, self-instruction, ignoring pain and passive strategies like catastrophizing, praying and hoping, and self-efficacy methods like overall reduction and control of pain. To test daily life restrictions, the ODI was used (0–20%: minimal disability, 21–40%: moderate disability, 41–60%: severe disability, 61–80%: crippling pain, 8–100% bed-bound patient or exaggeration of symptoms). The RSES detects with ten questions a tendency of reduced self-esteem. Lastly, the TS was used to determine fear of movement and (re)injury. Additionally, pain levels were evaluated by visual analogue scale (VAS), ranging from 1–10.

All questionnaires were completed in one session. Each session took about 45 min and the questionnaires were always completed in the same order as seen above. The patients did not criticize the length of the examinations.

### 2.3. Quantitative Sensory Testing (QST)

Thermal detection thresholds (cold and warm detection thresholds, CDT and WDT), thermal pain thresholds (cold and heat pain thresholds, CPT and HPT), mechanical detection threshold (MDT) and pressure pain threshold (PPT) were tested according to a standardized protocol published by Rolke, et al. [18]. These parameters were tested on the right hand and on the lower back (right side and left side). First, thermal detection thresholds were evaluated using the Thermal Sensory Analyzer II (TSA MEDOC Inc., Ramat Ishai, Israel), followed by thermal pain thresholds. The contact area of the thermode was 9 cm^2^ and the baseline temperature was 32 °C. Temperature was raised or lowered by 1 °C/s until thermal detection was indicated by the test person. The mean value of five consecutive measurements was calculated. Cut-off values for thermal pain were 0 °C and 50 °C, respectively.

Additionally, the mechanical detection threshold was measured using Von-Frey-Filaments with optical glass fibres (OptiHair2-Set, Marstock Nervtest, Deutschland). Furthermore, mechanical pain sensitivity data to blunt pressure was collected with a pressure algometer (FPK Algometer, Wagner Instruments, Greenwich, CT, USA). Contact area was 1 cm2 and stimuli were gradually increased by 50 kPa/s. Mean pressure pain thresholds were calculated using three consecutive measurements.

All tests were performed by one trained examiner only. Each session was about 45 min long and the tests were always performed in the same order. First, thermal detection thresholds, followed by thermal pain thresholds were evaluated. Lastly, mechanical detection and pressure pain thresholds were measured. Room temperature was constantly between 20 °C and 24 °C and the test persons were seated comfortably on a chair or lying down.

Quantitative sensory testing was performed at hospital admission, before hospital discharge and repeated after three to eight months. During this time, patients underwent conservative therapy or surgical therapy of a spinal infection. Results of QST were compared to a healthy control group (*n* = 20), as previously described [19]. These individuals were not constantly physically active and did not suffer from acute or chronic pain. The group consisted of 5 female and 15 male participants, with a mean age of 33.65 years (±10.18).

### 2.4. Statistical Analysis

Test results were evaluated using SPSS 27.0. Data (IBM SPSS Statistics Version 27, IBM Germany, Ehningen, Germany) were evaluated using descriptive statistics like mean and standard deviation. Differences among groups in QST as well as differences between the first, second and third QST sessions were calculated by non-parametric tests (Mann–Whitney U test, Wilcoxon test), because previous histograms revealed no statistical normal distribution among the collected values. Parametric statistical tests were used for the questionnaires (one-sample *t*-test) and the course of questionnaire-sessions (two-sample *t*-test). Connections between QST values and questionnaire results were evaluated with Spearman’s rank correlation coefficient. Values measured for the mean detection threshold (MDT) were logarithmic transformed, so a statistical calculation with a parametric test (one-sample *t*-test) was possible. The significance level was determined as *p* < 0.05.

## 3. Results

### 3.1. Patients’ Clinical Data

**Figure 1:**
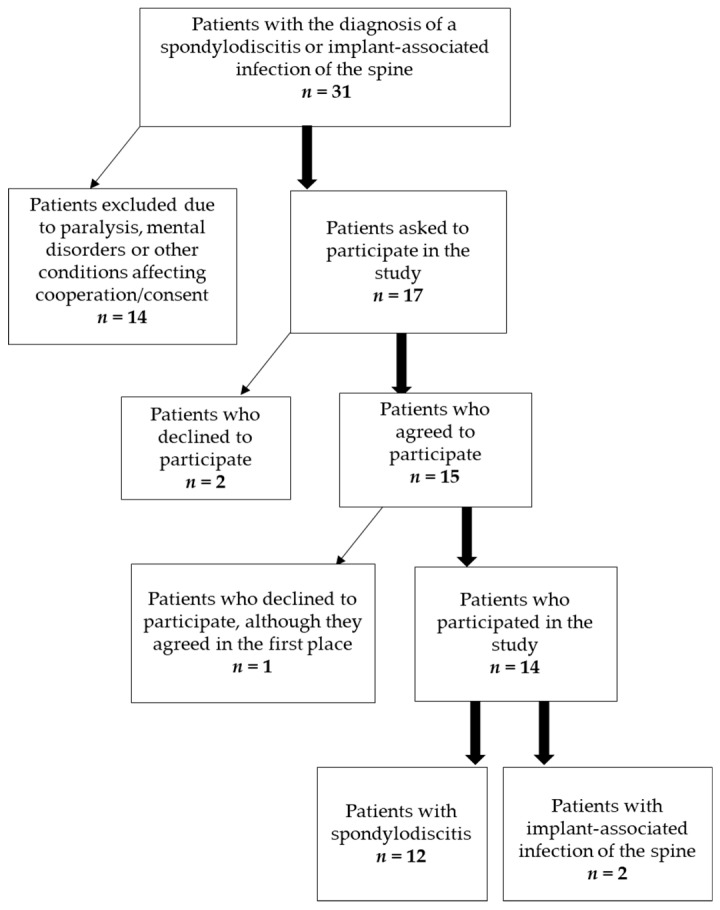
Patients recruited for this study.

**Figure 2:**
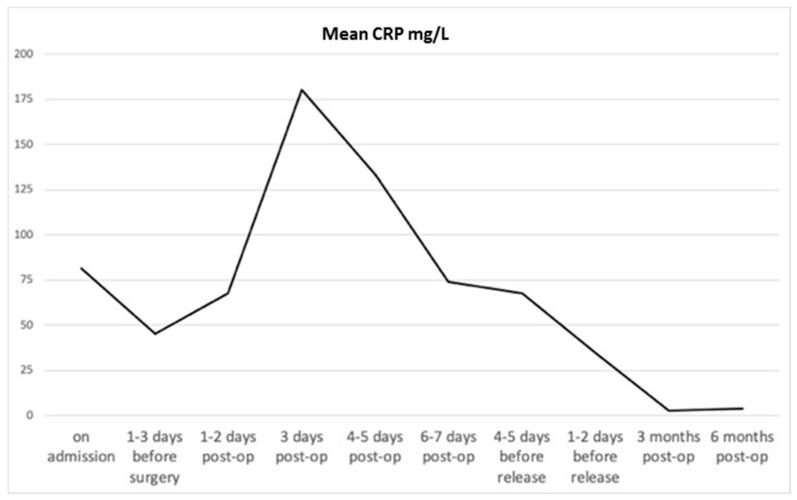
C-reactive protein measured in peripheral blood samples. Concentration was highest shortly after surgery and was within the normal range after 3 months. CRP: C-reactive protein.

Eight male and six female patients were included in this study (Figure 1). The average age of the patients was 66 years (±16.92). In-patient treatment lasted on average 45.3 days (±33.13). The patients complained of pain for 3.43 months (±2.77) (patients’ clinical data are shown in Table 1). Every patient was under pain medication, either NSAIDs (non-steroidal anti-inflammatory drug) only or a combination of World Health Organization (WHO) Level 1 and 3 drugs (Table 2). Following microbiological evaluation of tissue samples, bacteria were detected in 10 patients. Bacteria species and antibiotic treatment are summarized in Table 3. Over the course of time, CRP and white blood cell (WBC) count were measured in peripheral blood samples (Figure 2). CRP-concentration was highest shortly after surgery and was within the normal range after 3 months. White blood cell count did not show a significant increase during the course of the disease.

### 3.2. Questionnaires

**Figure 3:**
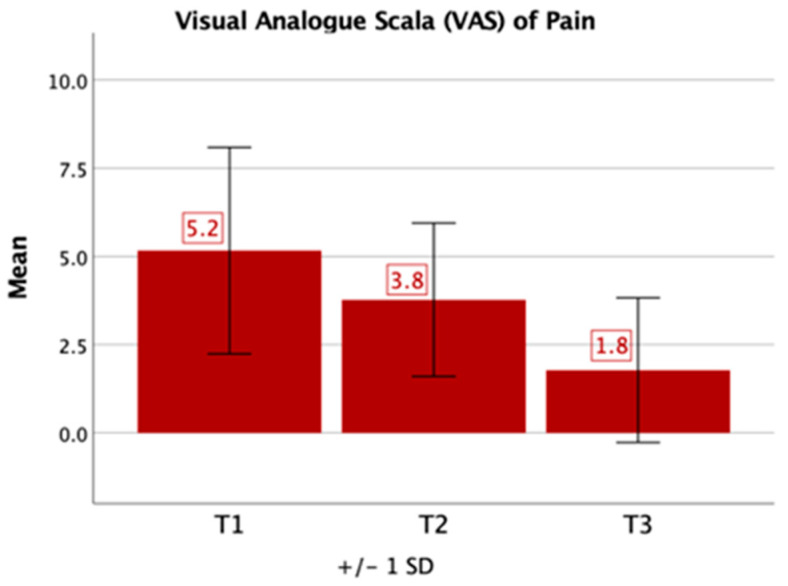
Time course visual analogue scale (VAS) of pain. Patients overall reported an improvement of pain symptoms during the course of the disease. SD: standard deviation.

In comparison to healthy control values, the values given on the VAS pain scale were significantly higher (T1: 5.2, T2: 3.8, T3: 1.8, *p* < 0.05). On the VAS pain scale (0–10), significant improvement of pain levels could be detected over the course of time between T1 and T3 (*p* = 0.005) (Figure 3).

In the PHQ, three patients (18.75%) at T1, three patients (18.75%) at T2 and one patient (6.25%) at T3 were above the cutoff for a depressive mood, whereas three patients (18.75%) at T1, one patient (6.25%) at T2 and none at T3 showed signs of anxiety. Six patients (37.5%) with spinal infections reported catastrophic thinking (PCS) at T1 and T2.

By means of the RQ, 2 patients (12.5%) related to statements consistent with a dismissive-avoidant and two patients (12.5%) with a fearful-avoidant attachment style at T1. At T2, only one patient (6.25%) related to a dismissive attachment style, while at T3 four patients (25%) related to this kind of attachment style.

**Figure 4:**
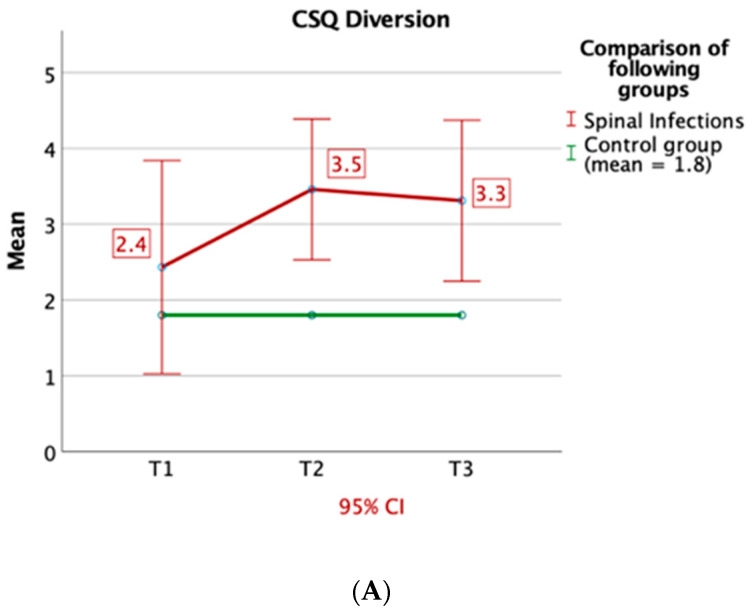

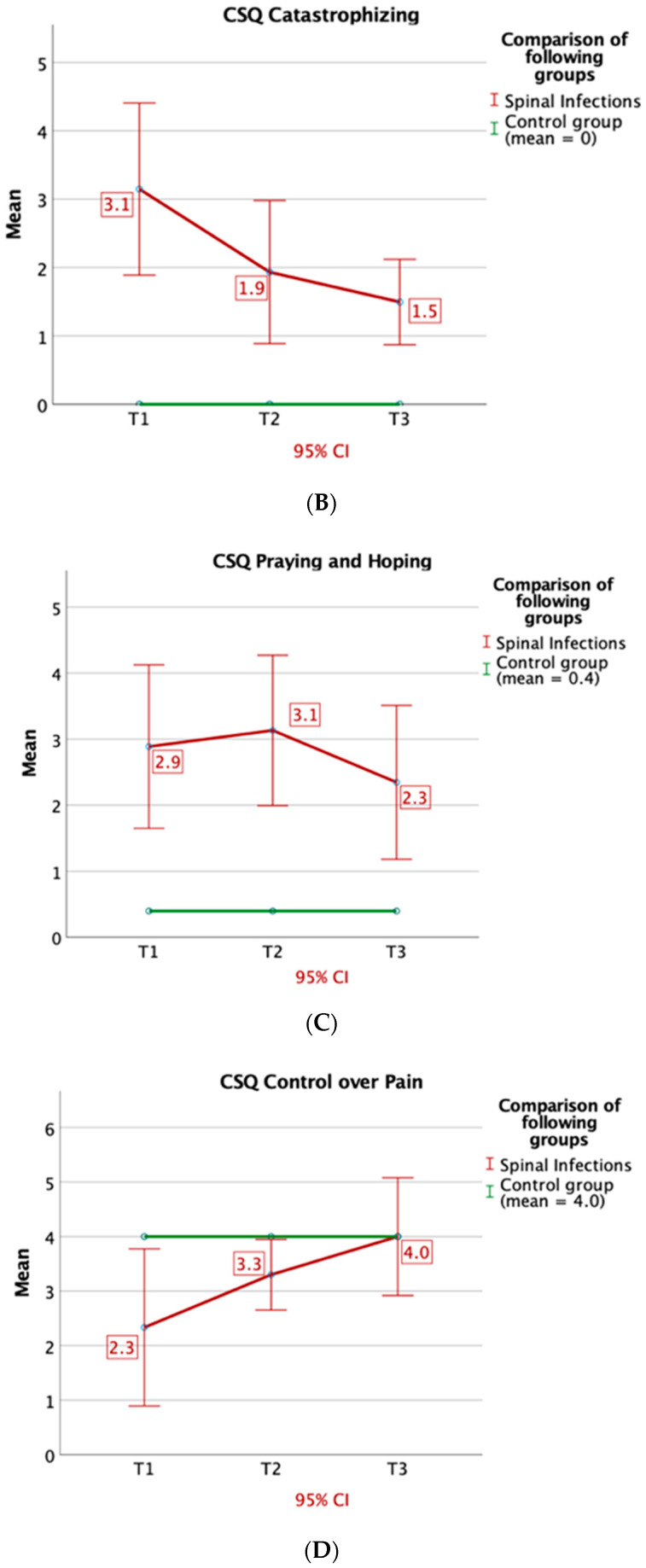
(**A**–**D**) Results of Coping Strategies Questionnaire (CSQ). The following results (compared to healthy individuals) are shown: active strategy, diversion (**A**), passive strategies, catastrophizing (**B**) and praying and hoping (**C**), and self-efficacy method, control over pain (**D**). Particularly during the inpatient stay (T1 and T2) patients with spinal infections turned to passive coping strategies, such as praying and hoping and catastrophizing. CI: confidence interval.

By means of the CSQ-D, patients with spinal infections experienced more catastrophic thinking (in T1-3: *p* = 0.001; *p* = 0.003; *p* = 0.001) and turned more to praying and hoping (in T1-3: *p* = 0.002; *p* = 0.001; *p* = 0.007). Furthermore, they used strategies like reinterpreting pain sensations (T1 and T3: *p* < 0.05) and diversion (T2: *p* = 0.004 and T3: *p* = 0.015). Also, the feeling of control over their pain was decreased at T1 and T2 (T1: *p* = 0.037 and T2: *p* = 0.045) (Figure 4A–D).

**Figure 5:**
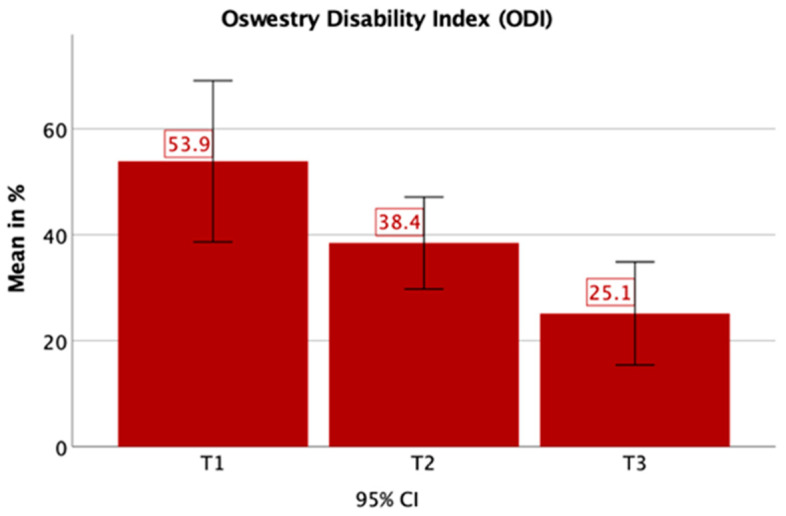
The Oswestry Disability Index (ODI) showed severe disablitiy at T1, and moderate disability at T2 and T3. There was a significant improvement from T1 to T3.

Using the ODI, averages of 54% (T1), 38% (T2) and 25% (T3) disability were determined, which all were significantly higher than a healthy individual (T1: *p* < 0.001, T2: *p* < 0.001, T3: *p* = 0.015). Still, they showed a significant improvement between T1 and T3 (*p* = 0.024). (Figure 5).

Patients with spinal infections showed significantly low self-esteem in the Rosenberg Self-esteem Scale (RSES T1: *p* = 0.001, T2: *p* = 0.029, T3: *p* = 0.038).

**Figure 6:**
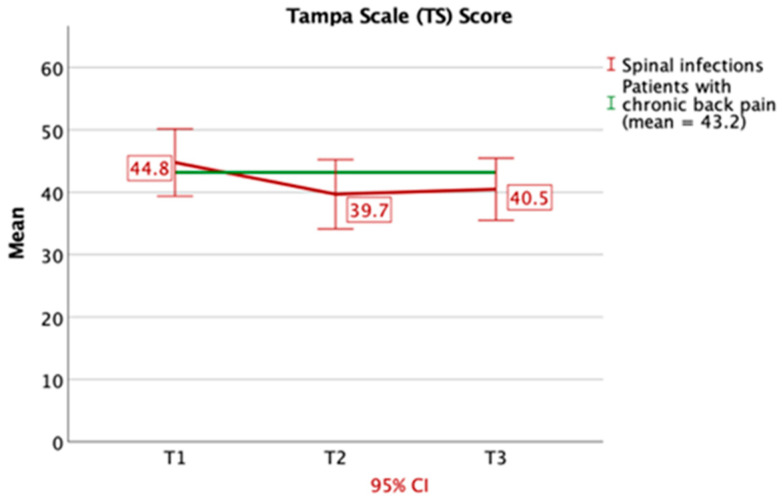
Results of the Tampa Scale are shown, which evaluates fear of movement and (re)-injury. Values of patients with spinal infections were comparable to patients suffering from chronic back pain at T1–T3. CI: confidence interval.

Concerning the Tampa Scale, which determines fear of movement, we ascertained averages of 44.8 (T1), 39.7 (T2) and 43.2 (T3), which all are very near the typical value of chronic back pain patients (43.2) (Figure 6).

In T1 and T2, there was a significant correlation between the test results of the PCS and Catastrophizing of the Coping Strategies Questionnaire (CSQ-D; T1: *p* = 0.014, T2: *p* = 0.001). Likewise, a significant association between the PCS and the ODI (T1: *p* = 0.041, T3: *p* = 0.002).

### 3.3. Quantitative Sensory Testing

**Figure 7:**
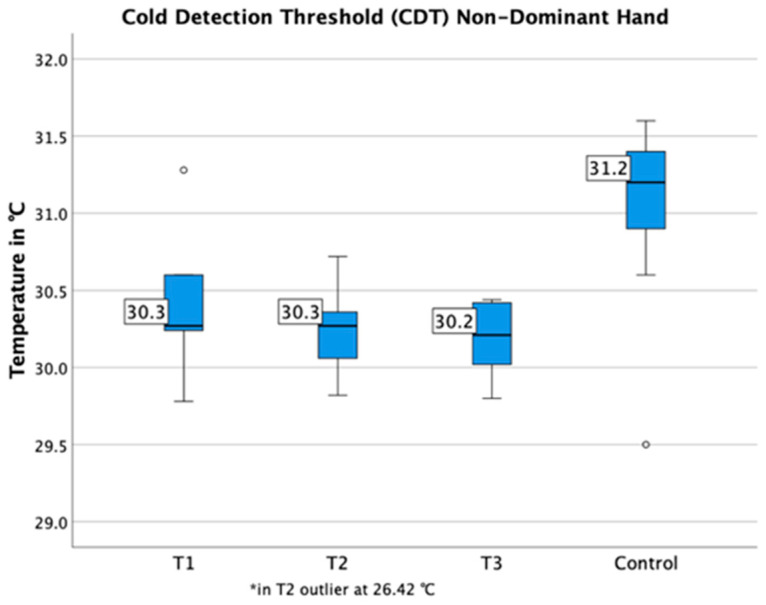
Cold detection thresholds of the control region (hand) are shown. Compared to healthy individuals, detection thresholds for cold temperature were decreased (T1–T3).

**Figure 8:**
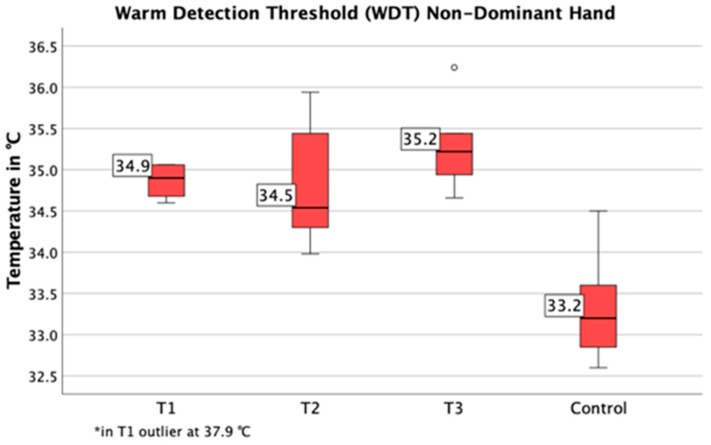
Warm detection thresholds of the control region (hand,) are shown. Compared to healthy individuals, detection thresholds for warm temperature were increased (T1–T3).

Compared to healthy individuals, the detection thresholds for cold temperatures (T1: *p* = 0.009, T2: *p* < 0.001, T3: *p* = 0.001) were decreased and detection thresholds for warm temperatures (T1-3: *p* < 0.001) were increased at the control region (hand) (Figure 7 and Figure 8). The cold detection threshold at the lower back was decreased as well (T1: *p* = 0.033, T2: *p* = 0.019, T3: *p* = 0.004). Additionally, in T2 patients with spinal infections were found to have significantly higher thresholds on their back for the detection of warm temperatures (WDT: right *p* = 0.003, left *p* = 0.001) and mechanical contact (MDT: right *p* = 0.018, left: *p* = 0.002) in comparison to the control region (hand).

**Figure 9:**
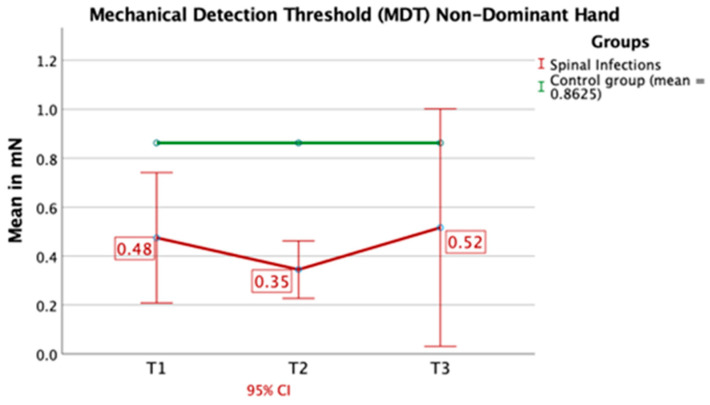
Mechanical detection thresholds of the control region (hand) were significantly lower compared to healthy individuals. CI: confidence interval.

The mechanical detection threshold of the control region (hand) was significantly lower over the course of time (T1: *p* = 0.023, T2: *p* < 0.001, T3: *p* = 0.046) (Figure 9).

## 4. Discussion

Spinal infections are particularly detrimental due to the fact that considerable destruction to the profile of the spine might cause neurological deficits and removal of an implant might lead to loss of correction, as well as stability of the spine [20]. Because of the frequently protracted course of the disease, patients often suffer from significant pain over a long period of time, which puts them at risk of pain chronification [21].

The aim of this study was to evaluate clinical data, risk factors for pain chronification, as well as coping strategies in patients suffering from infections of the spine.

The limitations of this study were that the number of patients included was not sufficient to further differentiate between test results of patients suffering from implant-associated infections of the spine (*n* = 2) and patients suffering from spondylodiscitis (*n* = 12). It could be of interest, if these two infectious groups show significant differences during the course of the disease. Furthermore, 12 patients received surgical treatment, predominately due to instability, and two patients underwent conservative treatment. It would be of further interest to evaluate these two groups separately, because there might be differences among patients regarding the risk of pain chronification and pain coping strategies. Another limitation of this study was that the time point T3 varied among patients (3–8 months) according to the individual course of the disease. Also, QST results were compared to healthy individuals. Patients of the control group were significantly younger than patients with spinal infections. It has been proposed that younger age might be associated with higher sensitivity to thermal stimuli [22]. On the other hand, Marouf et al. argue that age differences might not play a significant role in some pain regulatory processes [23]. The impact of age differences on QST results is, therefore, not yet fully understood.

Our results show that patients with spinal infections spent on average a month and a half in inpatient care. However, patients already suffered from persistent pain on average 3.4 months prior to admission. Despite the severity of the disease, patients showed significant improvement concerning the VAS pain level, which was reduced to an average of 1.8 at T3 and indicates that persistent pain is not a major issue in spinal infections. Additionally, patients reported moderate disability in the Oswestry Disability Index at T3, which also significantly improved over time and therefore suggests less limitation in everyday life compared to T1.

However, patients also reported a depressive or anxious mood in the PHQ, as well as catastrophic thinking in particular during inpatient treatment. On admission, merely two patients showed a fearful attachment style, which is typically associated with depression and catastrophizing, as well as the increased experience of negative affects and less flexible coping strategies [24]. Therefore, it seems unlikely that patients were prone to this kind of behavior all along and this, rather, indicates that mood changes were in fact triggered by the course of the infectious disease. This is supported by the fact that at the time of follow-up post-operatively, more patients presented a dismissive attachment style. These patients develop strategies that allow them to be compulsively independent because they feel uncomfortable trusting others. However, this attachment style is characterized by a positive attitude towards themselves, which then results in a characteristic self-reliance [24].

The CSQ showed in comparison to healthy individuals [25] that patients with spinal infections use active coping strategies, like reinterpreting pain sensations and diversion, but also focus on passive strategies, like catastrophizing and praying and hoping. Importantly, these coping strategies could be addressed by cognitive-behavioral therapy. During treatment active coping strategies are established and built upon, whereas the reduction of passive strategies is strived for [26,27]. It has been shown that passive coping strategies are predictive of poor treatment outcome, increased drug use, depression and fear of movement and re-injury [26,28]. With the feeling of helplessness, strategies of a passive nature are increasingly utilized. Patients who mainly use praying and hoping are more likely to express pain and are more functionally impaired. In general, the use of passive coping strategies can be associated with poorer adaptation to sensitive and emotional changes [29]. Therefore, intervention by means of cognitive-behavioral therapy could be beneficial for patients to establish active coping strategies early on.

The Tampa Scale (TS) is used to detect fear of movement and (re-)injury. The measured values of the TS in patients suffering from spinal infections were all similar to typical values measured in patients suffering from chronic back pain [30], even after discharge from inpatient treatment. All in all, this questionnaire represents a good predictor for the persistence of a pain-related impairment [31].

Furthermore, quantitative sensory testing was performed on all patients. Nociception is the perception of stimuli that potentially or actually harm the body. Nociceptors register these stimuli and they are then transmitted to the brain via afferent ascending pathways mainly in the spinothalamic tract, where they are located in parallel to the ascending pathways for thermal stimuli [32]. In thermal processes of the QST, Aδ and C-fibres are primarily stimulated [22]. Continuing pain correlates with thermal detection thresholds, therefore the sensitivity to non-painful stimuli could be suppressed by continuing pain [33]. In a study regarding patellofemoral pain syndrome, increased warm detection thresholds were found in the region of pain as well as in the control area. Decreased cold detection thresholds were also found in the painful test region [34]. Furthermore, also other musculoskeletal inflammatory diseases, such as osteoarthritis (OA), are accompanied by long-lasting pain as well. Quantitative sensory testing with a group of patients suffering from OA demonstrates abnormalities of the somatosensory perceptions in these patients. Here, they only show higher thermal thresholds at the local region of pain and not the control region, the forearm. Still, this also implies local thermal hypoaesthesia [35].

Our results are in line with this data. We also detected modified thermal thresholds at the control region (hand) and the test region (lower back), which indicates that patients suffering from spinal infections also possibly demonstrate a changed perception of pain.

Peripheral sensitization (primary hyperalgesia) indicates that nociceptors become more sensitive to stimuli. The excitation threshold of the nociceptors can be lowered to such an extent that non-noxic or non-painful stimuli are able to trigger action potentials. Moreover, sensitized nociceptors are characterised by a stronger response to supra-threshold stimuli. Central or spinal sensitization (secondary hyperalgesia) is characterized by a stronger response of neurons to stimuli from the diseased area and even to stimuli from adjacent, non-diseased areas [32]. This development from peripheral to central sensitization can be a typical form of pain chronification and we were interested whether this process could also be demonstrated in patients with spinal infections. However, this progression could not be shown by our QST data, because the detection thresholds for thermal pain did not differ significantly from healthy individuals.

## 5. Conclusions

In summary, patients with spinal infections showed significant improvement of pain levels and less limitation in everyday life over the course of the disease. Therefore, pain chronification did not occur in these patients. Furthermore, questionnaire results show distinctive features, such as the use of passive coping strategies, catastrophic thinking and fear of movement and injury. Therefore, patients might benefit from an interdisciplinary therapeutic approach, such as multidisciplinary pain therapy, which should be included in the treatment of spinal infections early on. Multidisciplinary pain therapy consists of somatic and psychotherapeutic procedures in combination with physical and psychological training programs, which can be adapted according to individual needs [36,37,38]. In this particular group of patients, active pain coping strategies should be emphasized, whereas passive coping strategies should be avoided. Furthermore, catastrophic thinking and fear of movement should be additionally addressed by means of psychological treatment. These techniques might help patients through the arduous treatment course of spinal infections.

## Figures and Tables

**Table 1:** Patients’ clinical data (mean values and standard deviations are shown).

Patients	Female	Male	Pain Duration in Months	Length of Inpatient Stay in Days	Age of Patients in Years
*n* = 14	*n* = 6	*n* = 8	3.43 (±2.77)	45.29 (±33.13)	66 (±16.92)

**Table 2:** Pain medication at the time of discharge from the hospital.

WHO Level 1	*n*	WHO Level 2	*n*	WHO Level 3	*n*	Co-Medication	
Metamizol 500 mg	7	On demand Tilidin/Naloxon 50 mg/4 mg	1	Hydromorphon 2–24 mg or on demand	2	Pregabalin 150 mg or 50 mg	2
Paracetamol 500 mg or 10 mg/mL i.v.	3			Oxycodon/Naloxon 40 mg/20 mg	1		
Ibuprofen 600 mg or 40 mg/mL	2			On demand Oxycodon 5 mg	1		
ASS 500 mg	1			Tapentadol 50 mg	1		

WHO: World Health Organization); ASS: Acetylsalicylic acid.

**Table 3:** Bacteria species detected in tissue samples and antibiotic therapy.

Bacteria Species	*n*	Antibiotics and Antimycotics	*n*	Overall Length of Intake in Days
*Staph. api*	4	Rifampicin	9	63.36 (±27.12)
*E. coli*	2	Ciprofloxacin	8	
*Candida albicans*	2	Cefuroxim	5	
*Staph. aureus*	2	Amoxicillin + Clavulansäure	5	
*Cutibacterium acnes*	2	Clindamycin	4	
*Pseudomonas aeruginosa*	1	Vancomycin	2	
*Strept. gallolyticus*	1	Fosfomycin	2	
*Strept. intermedius*	1	Fluconazol + Tazobactam	1	
None	5	And other penicillin, aminoglycoside, macrolides, cephalosporins, cabapenems		
